# Estimation of dynamical model parameters taking into account undetectable marker values

**DOI:** 10.1186/1471-2288-6-38

**Published:** 2006-08-01

**Authors:** Rodolphe Thiébaut, Jérémie Guedj, Hélène Jacqmin-Gadda, Geneviève Chêne, Pascale Trimoulet, Didier Neau, Daniel Commenges

**Affiliations:** 1 INSERM E0338 Biostatistics, Bordeaux 2 University, Bordeaux, France; 2 INSERM U593, Bordeaux 2 University, Bordeaux, France; 3 Department of virology, Bordeaux University Hospital, Bordeaux, France; 4 Department of infectious disease, Bordeaux University Hospital, Bordeaux, France

## Abstract

**Background:**

Mathematical models are widely used for studying the dynamic of infectious agents such as hepatitis C virus (HCV). Most often, model parameters are estimated using standard least-square procedures for each individual. Hierarchical models have been proposed in such applications. However, another issue is the left-censoring (undetectable values) of plasma viral load due to the lack of sensitivity of assays used for quantification. A method is proposed to take into account left-censored values for estimating parameters of non linear mixed models and its impact is demonstrated through a simulation study and an actual clinical trial of anti-HCV drugs.

**Methods:**

The method consists in a full likelihood approach distinguishing the contribution of observed and left-censored measurements assuming a lognormal distribution of the outcome. Parameters of analytical solution of system of differential equations taking into account left-censoring are estimated using standard software.

**Results:**

A simulation study with only 14% of measurements being left-censored showed that model parameters were largely biased (from -55% to +133% according to the parameter) with the exception of the estimate of initial outcome value when left-censored viral load values are replaced by the value of the threshold. When left-censoring was taken into account, the relative bias on fixed effects was equal or less than 2%. Then, parameters were estimated using the 100 measurements of HCV RNA available (with 12% of left-censored values) during the first 4 weeks following treatment initiation in the 17 patients included in the trial. Differences between estimates according to the method used were clinically significant, particularly on the death rate of infected cells. With the crude approach the estimate was 0.13 day^-1 ^(95% confidence interval [CI]: 0.11; 0.17) compared to 0.19 day^-1 ^(CI: 0.14; 0.26) when taking into account left-censoring. The relative differences between estimates of individual treatment efficacy according to the method used varied from 0.001% to 37%.

**Conclusion:**

We proposed a method that gives unbiased estimates if the assumed distribution is correct (e.g. lognormal) and that is easy to use with standard software.

## Background

Dynamical models based on system of differential equations have been successfully used for a better understanding of the pathogenesis of infectious diseases [1,2]. Two landmark papers appeared in 1995 demonstrating the high turnover of the human immunodeficiency virus (HIV) and infected CD4+ T lymphocytes cells [3,4]. Using such dynamical models, Neumann et al. [5] gave some insight in the effect of interferon based therapy used to treat patients infected by hepatitis C virus (HCV). Moreover, the estimate of the percentage of virus production blocked by the therapy is now widely used in this field [6-10] to evaluate the efficacy of treatment regimens in various contexts such as patients co-infected with HIV and HCV.

Although dynamical models parameters such as virus clearance or treatment efficacy are very useful, their estimation is most often performed for individual subjects separately. The limitations of such statistical approach as well as the interest of hierarchical models have already been underlined [11,12]. The main advantage of hierarchical models (also called mixed/random effects models) is their ability to estimate all parameters at the same time, using all available data even in case of unbalanced data, i.e. the number of measurements can vary from one patient to another. Parameters can be estimated using a Bayesian approach [13-15] or other approaches [16]. Another advantage working with analytical solutions of the system of differential equations is that standard softwares for non linear mixed models can be used [17].

Nonetheless, a major problem arises when using viral load data. The assays used to quantify HIV or HCV RNA are limited by a detection threshold that may lead to undetectable values when the true viral load is below this threshold. From a clinical point of view, the aim of any treatment is to reduce the viral load as much as possible [18]. Therefore, the practical definition of virological response is the occurrence of sustained undetectable values. The threshold of undetectable values is changing with the improvement of the assays for quantifying the viral load. When analysing viral load as a continuous variable, the left-censored measures are most often analyzed by replacing their value by an arbitrary value (e.g. threshold or half of the threshold). Although the sensitivity of the assays is improving, this limitation still persists and has already been underlined in the context of dynamical models [12,15]. Methods to take into account left-censored repeated measures in linear mixed models [19-21] or in non linear mixed models [15] have already been proposed. In this paper, we show how such an approach can be implemented using standard software in the case of non linear mixed models. Furthermore, we evaluate the impact of not taking into account undetectable values when studying HCV dynamics in the context of a phase II randomised clinical trial for the treatment of HCV infection in HIV co-infected patients.

## Methods

### Study example

The motivating study was a phase II randomised clinical trial evaluating the efficacy of pegylated-interferon (PEG-IFN)-α2a and Ribavirin (RBV) for the treatment of HCV infection among 17 HIV co-infected patients who had already been treated for HCV [22]. HCV RNA quantification was performed at least three times within the first 4 weeks (W): W0 (treatment initiation), W2 and W4. In 8 patients, blood samples were collected more intensively with additional measures at 6 hours (H6), H12, day 1 (D1), D2, D4, W1 and W3. Patients were followed until W72 for final evaluation of the virological response but the study of viral dynamics was restricted to the first 4 weeks because of model assumptions (see below). The concentration of plasma HCV RNA was determined using a quantitative reverse transcription polymerase chain reaction (RT-PCR) assay (Cobas Amplicor HCV Monitor Test, version 2.0; Roche Molecular Systems). The lower detection limit of this assay was 600 IU/mL, i.e. 2.78 log10 IU/mL. Of note, one international unit (IU) equals approximately 2.2 copies/mL.

### Mathematical model

The model used to estimate HCV dynamics was first described by Neumann et al. [5] with the following differential equations:

dTdt=s−μT−βVT     (1)
 MathType@MTEF@5@5@+=feaafiart1ev1aaatCvAUfKttLearuWrP9MDH5MBPbIqV92AaeXatLxBI9gBaebbnrfifHhDYfgasaacH8akY=wiFfYdH8Gipec8Eeeu0xXdbba9frFj0=OqFfea0dXdd9vqai=hGuQ8kuc9pgc9s8qqaq=dirpe0xb9q8qiLsFr0=vr0=vr0dc8meaabaqaciaacaGaaeqabaqabeGadaaakeaadaWcaaqaaiabdsgaKjabdsfaubqaaiabdsgaKjabdsha0baacqGH9aqpcqWGZbWCcqGHsisliiGacqWF8oqBcqWGubavcqGHsislcqWFYoGycqWGwbGvcqWGubavcaWLjaGaaCzcamaabmGabaGaeGymaedacaGLOaGaayzkaaaaaa@40FE@

dIdt=βVT−δI     (2)
 MathType@MTEF@5@5@+=feaafiart1ev1aaatCvAUfKttLearuWrP9MDH5MBPbIqV92AaeXatLxBI9gBaebbnrfifHhDYfgasaacH8akY=wiFfYdH8Gipec8Eeeu0xXdbba9frFj0=OqFfea0dXdd9vqai=hGuQ8kuc9pgc9s8qqaq=dirpe0xb9q8qiLsFr0=vr0=vr0dc8meaabaqaciaacaGaaeqabaqabeGadaaakeaadaWcaaqaaiabdsgaKjabdMeajbqaaiabdsgaKjabdsha0baacqGH9aqpiiGacqWFYoGycqWGwbGvcqWGubavcqGHsislcqWF0oazcqWGjbqscaWLjaGaaCzcamaabmGabaGaeGOmaidacaGLOaGaayzkaaaaaa@3E67@

dVdt=(1−ε)pI−cV     (3)
 MathType@MTEF@5@5@+=feaafiart1ev1aaatCvAUfKttLearuWrP9MDH5MBPbIqV92AaeXatLxBI9gBaebbnrfifHhDYfgasaacH8akY=wiFfYdH8Gipec8Eeeu0xXdbba9frFj0=OqFfea0dXdd9vqai=hGuQ8kuc9pgc9s8qqaq=dirpe0xb9q8qiLsFr0=vr0=vr0dc8meaabaqaciaacaGaaeqabaqabeGadaaakeaadaWcaaqaaiabdsgaKjabdAfawbqaaiabdsgaKjabdsha0baacqGH9aqpdaqadiqaaiabigdaXiabgkHiTGGaciab=v7aLbGaayjkaiaawMcaaiabdchaWjabdMeajjabgkHiTiabdogaJjabdAfawjaaxMaacaWLjaWaaeWaceaacqaIZaWmaiaawIcacaGLPaaaaaa@41D8@

where T is the number of target cells (i.e. hepatocytes), I the number of productively infected cells and V the plasma HCV viral load. Target cells are produced at rate s (per day) and die at rate μ. The number of cells which become infected per day is proportional to the number of circulating virions and available target cells with a proportionality constant β (infection rate). Infected cells die at rate δ per day. HCV virions are produced at a rate p per infected cells per day and are cleared at a rate c per day. In the present model, the HCV treatment is supposed to reduce the production of virions from infected cells by a fraction (1-ε). The possible effect of IFN as well as RBV on de novo rate of infection [5] or on infectivity by producing a fraction of non-infectious virions [23] have been discussed. For the purpose of this paper, we assumed only a combined effect of both drugs on production rate of new virions because this measure was the most widely used by other investigators [6-9].

When working on a short period of 2–4 weeks, it sounds reasonable to consider that the number of uninfected hepatocytes (T) remains constant (equal to the baseline value) because of the slow turnover of these cells [5]. Therefore, assuming a pre-treatment steady-state, the analytical solution of the equations (2) and (3) with T constant is:

V(t)=V0{Ae[−λ1(t−t0)]+(1−A)e[−λ2(t−t0)]}     (4)
 MathType@MTEF@5@5@+=feaafiart1ev1aaatCvAUfKttLearuWrP9MDH5MBPbIqV92AaeXatLxBI9gBaebbnrfifHhDYfgasaacH8akY=wiFfYdH8Gipec8Eeeu0xXdbba9frFj0=OqFfea0dXdd9vqai=hGuQ8kuc9pgc9s8qqaq=dirpe0xb9q8qiLsFr0=vr0=vr0dc8meaabaqaciaacaGaaeqabaqabeGadaaakeaacqWGwbGvcqGGOaakcqWG0baDcqGGPaqkcqGH9aqpcqWGwbGvdaWgaaWcbaGaeGimaadabeaakmaacmqabaGaemyqaeKaemyzau2aaWbaaSqabeaadaWadiqaaiabgkHiTGGaciab=T7aSnaaBaaameaacqaIXaqmaeqaaSWaaeWaceaacqWG0baDcqGHsislcqWG0baDdaWgaaadbaGaeGimaadabeaaaSGaayjkaiaawMcaaaGaay5waiaaw2faaaaakiabgUcaRmaabmGabaGaeGymaeJaeyOeI0IaemyqaeeacaGLOaGaayzkaaGaemyzau2aaWbaaSqabeaadaWadiqaaiabgkHiTiab=T7aSnaaBaaameaacqaIYaGmaeqaaSWaaeWaceaacqWG0baDcqGHsislcqWG0baDdaWgaaadbaGaeGimaadabeaaaSGaayjkaiaawMcaaaGaay5waiaaw2faaaaaaOGaay5Eaiaaw2haaiaaxMaacaWLjaWaaeWaceaacqaI0aanaiaawIcacaGLPaaaaaa@5C4C@

for t>t_0_, where

*λ*_1 _= {(*c *+ *δ*) + (c−δ)2+4(1−ε)cδ
 MathType@MTEF@5@5@+=feaafiart1ev1aaatCvAUfKttLearuWrP9MDH5MBPbIqV92AaeXatLxBI9gBaebbnrfifHhDYfgasaacH8akY=wiFfYdH8Gipec8Eeeu0xXdbba9frFj0=OqFfea0dXdd9vqai=hGuQ8kuc9pgc9s8qqaq=dirpe0xb9q8qiLsFr0=vr0=vr0dc8meaabaqaciaacaGaaeqabaqabeGadaaakeaadaGcaaqaamaabmGabaGaem4yamMaeyOeI0ccciGae8hTdqgacaGLOaGaayzkaaWaaWbaaSqabeaacqaIYaGmaaGccqGHRaWkcqaI0aandaqadiqaaiabigdaXiabgkHiTiab=v7aLbGaayjkaiaawMcaaiabdogaJjab=r7aKbWcbeaaaaa@3D34@}, *λ*_2 _= {(*c *+ *δ*) - (c−δ)2+4(1−ε)cδ
 MathType@MTEF@5@5@+=feaafiart1ev1aaatCvAUfKttLearuWrP9MDH5MBPbIqV92AaeXatLxBI9gBaebbnrfifHhDYfgasaacH8akY=wiFfYdH8Gipec8Eeeu0xXdbba9frFj0=OqFfea0dXdd9vqai=hGuQ8kuc9pgc9s8qqaq=dirpe0xb9q8qiLsFr0=vr0=vr0dc8meaabaqaciaacaGaaeqabaqabeGadaaakeaadaGcaaqaamaabmGabaGaem4yamMaeyOeI0ccciGae8hTdqgacaGLOaGaayzkaaWaaWbaaSqabeaacqaIYaGmaaGccqGHRaWkcqaI0aandaqadiqaaiabigdaXiabgkHiTiab=v7aLbGaayjkaiaawMcaaiabdogaJjab=r7aKbWcbeaaaaa@3D34@} and A=(ε c−λ2)(λ1−λ2)
 MathType@MTEF@5@5@+=feaafiart1ev1aaatCvAUfKttLearuWrP9MDH5MBPbIqV92AaeXatLxBI9gBaebbnrfifHhDYfgasaacH8akY=wiFfYdH8Gipec8Eeeu0xXdbba9frFj0=OqFfea0dXdd9vqai=hGuQ8kuc9pgc9s8qqaq=dirpe0xb9q8qiLsFr0=vr0=vr0dc8meaabaqaciaacaGaaeqabaqabeGadaaakeaacqWGbbqqcqGH9aqpdaWcaaqaamaabmGabaacciGae8xTduMaeeiiaaIaem4yamMaeyOeI0Iae83UdW2aaSbaaSqaaiabikdaYaqabaaakiaawIcacaGLPaaaaeaadaqadiqaaiab=T7aSnaaBaaaleaacqaIXaqmaeqaaOGaeyOeI0Iae83UdW2aaSbaaSqaaiabikdaYaqabaaakiaawIcacaGLPaaaaaaaaa@4004@. The viral decay is assumed to begin at *t*_0 _= 0.25 day (6 hours), corresponding to the drugs pharmacokinetics [5].

### Hierarchical formulation

The previous notations do not account for patient/measurement level. Most often parameters of such models are estimated patient by patient assuming Gaussian, homoskedastic measurement error. A more valid and powerful approach is based on a hierarchical formulation of the model [11] that can distinguish at least two levels of variation. Hence, for the j^th ^measurement of a subject i performed at a time t_ij_:

- Stage 1: intra-patient variation

*y*_*ij *_= log_10_(*V*(*t*_*ij*_, θ_*i*_)) + *e*

with ei∼N(0,σe2Ini)
 MathType@MTEF@5@5@+=feaafiart1ev1aaatCvAUfKttLearuWrP9MDH5MBPbIqV92AaeXatLxBI9gBaebbnrfifHhDYfgasaacH8akY=wiFfYdH8Gipec8Eeeu0xXdbba9frFj0=OqFfea0dXdd9vqai=hGuQ8kuc9pgc9s8qqaq=dirpe0xb9q8qiLsFr0=vr0=vr0dc8meaabaqaciaacaGaaeqabaqabeGadaaakeaacqWGLbqzdaWgaaWcbaGaemyAaKgabeaakiablYJi6iabd6eaonaabmGabaGaeGimaaJaeiilaWccciGae83Wdm3aa0baaSqaaiabdwgaLbqaaiabikdaYaaakiabdMeajnaaBaaaleaacqWGUbGBdaWgaaadbaGaemyAaKgabeaaaSqabaaakiaawIcacaGLPaaaaaa@3DC6@

The outcome is the logarithm (base 10) of the true viral load (function of t_ij _and θ_i_, the p-vector of model parameters) plus a Gaussian measurement error e. I_ni _is a identity matrix of dimension n_i _× n_i_, n_i _being the number of measurements available for the subject i.

- Stage 2: inter-patient variation

θ_*i *_= θ + *γ*_*i*_

with *γ*_*i *_~ *MVN*(0, *D*)

θ = [V_0_, ε, δ, c] is the p-vector of average (fixed) effect in the whole study population and γ_*i *_is a q-vector (q ≤ p) of random effects for correcting θ for each subject (random effect). Actually, θ is a log-transformation of original parameters that have several advantages including a positivity constraint for original parameters. Random effects γ_*i *_were assumed to be normally distributed with a variance-covariance matrix D. θ_*i *_are estimated through Empirical Bayes estimates.

### Model likelihood

As presented in more details elsewhere [24], the method proposed to take into account left-censored values when estimating parameters is to maximise a full likelihood distinguishing the contribution of observed measures (Yij0
 MathType@MTEF@5@5@+=feaafiart1ev1aaatCvAUfKttLearuWrP9MDH5MBPbIqV92AaeXatLxBI9gBaebbnrfifHhDYfgasaacH8akY=wiFfYdH8Gipec8Eeeu0xXdbba9frFj0=OqFfea0dXdd9vqai=hGuQ8kuc9pgc9s8qqaq=dirpe0xb9q8qiLsFr0=vr0=vr0dc8meaabaqaciaacaGaaeqabaqabeGadaaakeaacqWGzbqwdaqhaaWcbaGaemyAaKMaemOAaOgabaGaeGimaadaaaaa@31BA@ for j = 1,...ni0
 MathType@MTEF@5@5@+=feaafiart1ev1aaatCvAUfKttLearuWrP9MDH5MBPbIqV92AaeXatLxBI9gBaebbnrfifHhDYfgasaacH8akY=wiFfYdH8Gipec8Eeeu0xXdbba9frFj0=OqFfea0dXdd9vqai=hGuQ8kuc9pgc9s8qqaq=dirpe0xb9q8qiLsFr0=vr0=vr0dc8meaabaqaciaacaGaaeqabaqabeGadaaakeaacqWGUbGBdaqhaaWcbaGaemyAaKgabaGaeGimaadaaaaa@3087@) and left-censored measures (Yijc
 MathType@MTEF@5@5@+=feaafiart1ev1aaatCvAUfKttLearuWrP9MDH5MBPbIqV92AaeXatLxBI9gBaebbnrfifHhDYfgasaacH8akY=wiFfYdH8Gipec8Eeeu0xXdbba9frFj0=OqFfea0dXdd9vqai=hGuQ8kuc9pgc9s8qqaq=dirpe0xb9q8qiLsFr0=vr0=vr0dc8meaabaqaciaacaGaaeqabaqabeGadaaakeaacqWGzbqwdaqhaaWcbaGaemyAaKMaemOAaOgabaGaem4yamgaaaaa@321B@ for j = 1,...nic
 MathType@MTEF@5@5@+=feaafiart1ev1aaatCvAUfKttLearuWrP9MDH5MBPbIqV92AaeXatLxBI9gBaebbnrfifHhDYfgasaacH8akY=wiFfYdH8Gipec8Eeeu0xXdbba9frFj0=OqFfea0dXdd9vqai=hGuQ8kuc9pgc9s8qqaq=dirpe0xb9q8qiLsFr0=vr0=vr0dc8meaabaqaciaacaGaaeqabaqabeGadaaakeaacqWGUbGBdaqhaaWcbaGaemyAaKgabaGaem4yamgaaaaa@30E8@) of viral load. The likelihood can be written:

L(θ)=∏i=1N[∫Rq{∏j=1ni0fYij0|γi(Yij0|γi=u)}{∏j=ni0+1nicFYijc|γi(Yijc|γi=u)}fγi(u)du]     (5)
 MathType@MTEF@5@5@+=feaafiart1ev1aaatCvAUfKttLearuWrP9MDH5MBPbIqV92AaeXatLxBI9gBaebbnrfifHhDYfgasaacH8akY=wiFfYdH8Gipec8Eeeu0xXdbba9frFj0=OqFfea0dXdd9vqai=hGuQ8kuc9pgc9s8qqaq=dirpe0xb9q8qiLsFr0=vr0=vr0dc8meaabaqaciaacaGaaeqabaqabeGadaaakeaacqWGmbatcqGGOaakiiGacqWF4oqCcqGGPaqkcqGH9aqpdaqeWbqaamaadmGabaWaa8quaeaadaGadeqaamaarahabaGaemOzay2aaSbaaSqaamaaeiGabaGaemywaK1aa0baaWqaaiabdMgaPjabdQgaQbqaaiabicdaWaaaaSGaayjcSdGae83SdC2aaSbaaWqaaiabdMgaPbqabaaaleqaaaqaaiabdQgaQjabg2da9iabigdaXaqaaiabd6gaUnaaBaaameaacqWGPbqAcqaIWaamaeqaaaqdcqGHpis1aOWaaeWaceaadaabciqaaiabdMfaznaaDaaaleaacqWGPbqAcqWGQbGAaeaacqaIWaamaaaakiaawIa7aiab=n7aNnaaBaaaleaacqWGPbqAaeqaaOGaeyypa0JaemyDauhacaGLOaGaayzkaaaacaGL7bGaayzFaaWaaiWabeaadaqeWbqaaiabdAeagnaaBaaaleaadaabciqaaiabdMfaznaaDaaameaacqWGPbqAcqWGQbGAaeaacqWGJbWyaaaaliaawIa7aiab=n7aNnaaBaaameaacqWGPbqAaeqaaaWcbeaaaeaacqWGQbGAcqGH9aqpcqWGUbGBdaWgaaadbaGaemyAaKMaeGimaadabeaaliabgUcaRiabigdaXaqaaiabd6gaUnaaBaaameaacqWGPbqAcqWGJbWyaeqaaaqdcqGHpis1aOWaaeWaceaadaabciqaaiabdMfaznaaDaaaleaacqWGPbqAcqWGQbGAaeaacqWGJbWyaaaakiaawIa7aiab=n7aNnaaBaaaleaacqWGPbqAaeqaaOGaeyypa0JaemyDauhacaGLOaGaayzkaaaacaGL7bGaayzFaaGaemOzay2aaSbaaSqaaiab=n7aNnaaBaaameaacqWGPbqAaeqaaaWcbeaakiabcIcaOiabdwha1jabcMcaPiabdsgaKjabdwha1bWcbaGaemOuai1aaWbaaWqabeaacqWGXbqCaaaaleqaniabgUIiYdaakiaawUfacaGLDbaacaWLjaGaaCzcamaabmGabaGaeGynaudacaGLOaGaayzkaaaaleaacqWGPbqAcqGH9aqpcqaIXaqmaeaacqWGobGta0Gaey4dIunaaaa@9DDB@

with fYijc|γi
 MathType@MTEF@5@5@+=feaafiart1ev1aaatCvAUfKttLearuWrP9MDH5MBPbIqV92AaeXatLxBI9gBaebbnrfifHhDYfgasaacH8akY=wiFfYdH8Gipec8Eeeu0xXdbba9frFj0=OqFfea0dXdd9vqai=hGuQ8kuc9pgc9s8qqaq=dirpe0xb9q8qiLsFr0=vr0=vr0dc8meaabaqaciaacaGaaeqabaqabeGadaaakeaacqWGMbGzdaWgaaWcbaWaaqGaceaacqWGzbqwdaqhaaadbaGaemyAaKMaemOAaOgabaGaem4yamgaaaWccaGLiWoaiiGacqWFZoWzdaWgaaadbaGaemyAaKgabeaaaSqabaaaaa@3881@ being the univariate normal density of Yij0
 MathType@MTEF@5@5@+=feaafiart1ev1aaatCvAUfKttLearuWrP9MDH5MBPbIqV92AaeXatLxBI9gBaebbnrfifHhDYfgasaacH8akY=wiFfYdH8Gipec8Eeeu0xXdbba9frFj0=OqFfea0dXdd9vqai=hGuQ8kuc9pgc9s8qqaq=dirpe0xb9q8qiLsFr0=vr0=vr0dc8meaabaqaciaacaGaaeqabaqabeGadaaakeaacqWGzbqwdaqhaaWcbaGaemyAaKMaemOAaOgabaGaeGimaadaaaaa@31BA@ given the random effects *γ*_i _and FYijc|γi
 MathType@MTEF@5@5@+=feaafiart1ev1aaatCvAUfKttLearuWrP9MDH5MBPbIqV92AaeXatLxBI9gBaebbnrfifHhDYfgasaacH8akY=wiFfYdH8Gipec8Eeeu0xXdbba9frFj0=OqFfea0dXdd9vqai=hGuQ8kuc9pgc9s8qqaq=dirpe0xb9q8qiLsFr0=vr0=vr0dc8meaabaqaciaacaGaaeqabaqabeGadaaakeaacqWGgbGrdaWgaaWcbaWaaqGaceaacqWGzbqwdaqhaaadbaGaemyAaKMaemOAaOgabaGaem4yamgaaaWccaGLiWoaiiGacqWFZoWzdaWgaaadbaGaemyAaKgabeaaaSqabaaaaa@3841@ is the cumulative distribution function of the normal distribution of Yijc
 MathType@MTEF@5@5@+=feaafiart1ev1aaatCvAUfKttLearuWrP9MDH5MBPbIqV92AaeXatLxBI9gBaebbnrfifHhDYfgasaacH8akY=wiFfYdH8Gipec8Eeeu0xXdbba9frFj0=OqFfea0dXdd9vqai=hGuQ8kuc9pgc9s8qqaq=dirpe0xb9q8qiLsFr0=vr0=vr0dc8meaabaqaciaacaGaaeqabaqabeGadaaakeaacqWGzbqwdaqhaaWcbaGaemyAaKMaemOAaOgabaGaem4yamgaaaaa@321B@ given the random effects. The calculation of this likelihood leads to the integration over *u *= *u*_1_,*u*_2_,...*u*_*q*_, that is a multiple integral of dimension q. Therefore, with this method, rather than imputing a fixed value of undetectable viral load, one assumes that left-censored values are completing the Gaussian distribution of Y_i_. A crude approach assumes that left-censored values contribute like observed values, being equal to the value of the threshold or any other given value. In this case, the likelihood is simply:

L(θ)=∏i=1N[∫Rq{∏j=1ni0+nicfYij|γi(Yij|γi=u)}fγi(u)du]     (6)
 MathType@MTEF@5@5@+=feaafiart1ev1aaatCvAUfKttLearuWrP9MDH5MBPbIqV92AaeXatLxBI9gBaebbnrfifHhDYfgasaacH8akY=wiFfYdH8Gipec8Eeeu0xXdbba9frFj0=OqFfea0dXdd9vqai=hGuQ8kuc9pgc9s8qqaq=dirpe0xb9q8qiLsFr0=vr0=vr0dc8meaabaqaciaacaGaaeqabaqabeGadaaakeaacqWGmbatcqGGOaakiiGacqWF4oqCcqGGPaqkcqGH9aqpdaqeWbqaamaadmGabaWaa8quaeaadaGadeqaamaarahabaGaemOzay2aaSbaaSqaamaaeiGabaGaemywaK1aaSbaaWqaaiabdMgaPjabdQgaQbqabaaaliaawIa7aiab=n7aNnaaBaaameaacqWGPbqAaeqaaaWcbeaaaeaacqWGQbGAcqGH9aqpcqaIXaqmaeaacqWGUbGBdaWgaaadbaGaemyAaKMaeGimaadabeaaliabgUcaRiabd6gaUnaaBaaameaacqWGPbqAcqWGJbWyaeqaaaqdcqGHpis1aOWaaeWaceaadaabciqaaiabdMfaznaaBaaaleaacqWGPbqAcqWGQbGAaeqaaaGccaGLiWoacqWFZoWzdaWgaaWcbaGaemyAaKgabeaakiabg2da9iabdwha1bGaayjkaiaawMcaaaGaay5Eaiaaw2haaiabdAgaMnaaBaaaleaacqWFZoWzdaWgaaadbaGaemyAaKgabeaaaSqabaGccqGGOaakcqWG1bqDcqGGPaqkcqWGKbazcqWG1bqDaSqaaiabdkfasnaaCaaameqabaGaemyCaehaaaWcbeqdcqGHRiI8aaGccaGLBbGaayzxaaaaleaacqWGPbqAcqGH9aqpcqaIXaqmaeaacqWGobGta0Gaey4dIunakiaaxMaacaWLjaWaaeWaceaacqaI2aGnaiaawIcacaGLPaaaaaa@76A8@

### Algorithm and implementation

The maximisation of the likelihood function can be performed with standard software such as NLMIXED in SAS^® ^[24]. Using this procedure, the default algorithm is a quasi-newton algorithm and the calculation of the multiple integral is performed by adaptative quadrature. An example of code used for this paper is provided in appendix.

### Simulation study

Simulations were performed to compare the bias on parameter estimates when taking into account left-censoring or not. Using the analytical solution (4) and allowing a random individual variation for the initial viral load and treatment efficacy, parameters to estimate were: ⌊*V*_0_, ε, δ *c*, σγ02
 MathType@MTEF@5@5@+=feaafiart1ev1aaatCvAUfKttLearuWrP9MDH5MBPbIqV92AaeXatLxBI9gBaebbnrfifHhDYfgasaacH8akY=wiFfYdH8Gipec8Eeeu0xXdbba9frFj0=OqFfea0dXdd9vqai=hGuQ8kuc9pgc9s8qqaq=dirpe0xb9q8qiLsFr0=vr0=vr0dc8meaabaqaciaacaGaaeqabaqabeGadaaakeaaiiGacqWFdpWCdaqhaaWcbaGae83SdC2aaSbaaWqaaiabicdaWaqabaaaleaacqaIYaGmaaaaaa@325D@, σγ12
 MathType@MTEF@5@5@+=feaafiart1ev1aaatCvAUfKttLearuWrP9MDH5MBPbIqV92AaeXatLxBI9gBaebbnrfifHhDYfgasaacH8akY=wiFfYdH8Gipec8Eeeu0xXdbba9frFj0=OqFfea0dXdd9vqai=hGuQ8kuc9pgc9s8qqaq=dirpe0xb9q8qiLsFr0=vr0=vr0dc8meaabaqaciaacaGaaeqabaqabeGadaaakeaaiiGacqWFdpWCdaqhaaWcbaGae83SdC2aaSbaaWqaaiabigdaXaqabaaaleaacqaIYaGmaaaaaa@325F@, σe2
 MathType@MTEF@5@5@+=feaafiart1ev1aaatCvAUfKttLearuWrP9MDH5MBPbIqV92AaeXatLxBI9gBaebbnrfifHhDYfgasaacH8akY=wiFfYdH8Gipec8Eeeu0xXdbba9frFj0=OqFfea0dXdd9vqai=hGuQ8kuc9pgc9s8qqaq=dirpe0xb9q8qiLsFr0=vr0=vr0dc8meaabaqaciaacaGaaeqabaqabeGadaaakeaaiiGacqWFdpWCdaqhaaWcbaGaemyzaugabaGaeGOmaidaaaaa@30E8@⌋

with V_0i _= V_0 _+ γ_0i_, ε_i _= ε + γ_1i _and [γ0iγ1i]∼N([00],[σγ0200σγ12])
 MathType@MTEF@5@5@+=feaafiart1ev1aaatCvAUfKttLearuWrP9MDH5MBPbIqV92AaeXatLxBI9gBaebbnrfifHhDYfgasaacH8akY=wiFfYdH8Gipec8Eeeu0xXdbba9frFj0=OqFfea0dXdd9vqai=hGuQ8kuc9pgc9s8qqaq=dirpe0xb9q8qiLsFr0=vr0=vr0dc8meaabaqaciaacaGaaeqabaqabeGadaaakeaadaWadiqaauaabeqaceaaaeaaiiGacqWFZoWzdaWgaaWcbaGaeGimaaJaemyAaKgabeaaaOqaaiab=n7aNnaaBaaaleaacqaIXaqmcqWGPbqAaeqaaaaaaOGaay5waiaaw2faaiablYJi6iabd6eaonaabmGabaWaamWaceaafaqabeGabaaabaGaeGimaadabaGaeGimaadaaaGaay5waiaaw2faaiabcYcaSmaadmGabaqbaeqabiGaaaqaaiab=n8aZnaaDaaaleaacqWFZoWzdaWgaaadbaGaeGimaadabeaaaSqaaiabikdaYaaaaOqaaiabicdaWaqaaiabicdaWaqaaiab=n8aZnaaDaaaleaacqWFZoWzdaWgaaadbaGaeGymaedabeaaaSqaaiabikdaYaaaaaaakiaawUfacaGLDbaaaiaawIcacaGLPaaaaaa@4ED3@

To constraint parameters to be in the correct range, estimations were performed on transformed parameters (for the study on real data, as well) using a logarithm function for δ, c and logit function for ε (bounding ε between 0 and 1). In the simulation study, we fixed t_0 _= 0 but results were similar with t_0 _= 0.25.

Values for model parameters were defined according to the results reported in the literature of HCV dynamics [6]. In our application where patients were previously treated and dually infected by HIV and HCV, the estimate of treatment efficacy is less than those usually reported in naïve patients mono-infected with HCV [5].

The steps followed for the simulations were:

1) Sample V_0i _= V_0 _+γ_0i _and ε_i _= ε + γ_1i _for a subject i

2) Simulate the differential equations (1)-(3) model and keep measures at the time points: keep measures at H0, H6, H12, D1, D2, D4, W1, W2, W3 and W4. Left-censor measures below 2.78 IU/mL.

3) Repeat N times (for N = 20 subjects) steps 1 and 2

4) Estimate parameters with (5) when taking into account left-censoring and with usual likelihood (6) replacing left-censored values by the value of the threshold, i.e. 2.78 IU/mL.

5) Calculate the relative bias for each parameter RB = 100*(estimate-true value)/true value

6) Repeat 1000 times steps 1 to 5 and average the relative biases

## Results

### Simulation study

Results of the simulations are shown in Table 1. On average, 14% of simulated measures of HIV RNA were left-censored when the treatment efficacy was ε = 80%. Crude estimates provided by standard likelihood (6) maximisation, replacing left-censored values by the value of the threshold, were dramatically biased with the exception of *V*_0_. In particular, with only 14% of left-censored measures, infected cells death rate δ and clearance of virus c were underestimated by 55 and 45 percent, respectively. Treatment efficacy (ε) was overestimated by 16%. Variances of random effects were also significantly biased: +20% and -19% for random effects on V_0_, and ε, respectively. The residual variance was overestimated (+133%).

**Table 1:** Relative bias of model parameters using non linear mixed models taking into account left-censored (undetectable) values (corrected) or not (crude) with simulated data. N = 20 patients, 1000 simulations, 14% left-censored measures in average.

Parameter and true value		Crude estimate		Corrected estimate	
		Estimates	Relative bias (%)	Estimates	Relative bias (%)

Fixed effects					
*V*_0_	6.16 log_10 _IU/mL	6.09	-1.1	6.16	-0.021
δ	0.40 day^-1^	0.18	-55.2	0.40	+0.78
c	2.00 day^-1^	1.10	-44.8	2.03	+1.5
ε	0.80	0.93	+15.8	0.79	-1.3

Variances					
σγ02 MathType@MTEF@5@5@+=feaafiart1ev1aaatCvAUfKttLearuWrP9MDH5MBPbIqV92AaeXatLxBI9gBaebbnrfifHhDYfgasaacH8akY=wiFfYdH8Gipec8Eeeu0xXdbba9frFj0=OqFfea0dXdd9vqai=hGuQ8kuc9pgc9s8qqaq=dirpe0xb9q8qiLsFr0=vr0=vr0dc8meaabaqaciaacaGaaeqabaqabeGadaaakeaaiiGacqWFdpWCdaqhaaWcbaGae83SdC2aaSbaaWqaaiabicdaWaqabaaaleaacqaIYaGmaaaaaa@325D@	0.49	0.59	+20.3	0.46	-6.4
σγ12 MathType@MTEF@5@5@+=feaafiart1ev1aaatCvAUfKttLearuWrP9MDH5MBPbIqV92AaeXatLxBI9gBaebbnrfifHhDYfgasaacH8akY=wiFfYdH8Gipec8Eeeu0xXdbba9frFj0=OqFfea0dXdd9vqai=hGuQ8kuc9pgc9s8qqaq=dirpe0xb9q8qiLsFr0=vr0=vr0dc8meaabaqaciaacaGaaeqabaqabeGadaaakeaaiiGacqWFdpWCdaqhaaWcbaGae83SdC2aaSbaaWqaaiabigdaXaqabaaaleaacqaIYaGmaaaaaa@325F@	2.69	2.18	-18.8	2.31	-13.9
σe2 MathType@MTEF@5@5@+=feaafiart1ev1aaatCvAUfKttLearuWrP9MDH5MBPbIqV92AaeXatLxBI9gBaebbnrfifHhDYfgasaacH8akY=wiFfYdH8Gipec8Eeeu0xXdbba9frFj0=OqFfea0dXdd9vqai=hGuQ8kuc9pgc9s8qqaq=dirpe0xb9q8qiLsFr0=vr0=vr0dc8meaabaqaciaacaGaaeqabaqabeGadaaakeaaiiGacqWFdpWCdaqhaaWcbaGaemyzaugabaGaeGOmaidaaaaa@30E8@	0.04	0.09	+133.2	0.039	-2.4

When taking into account left-censoring, the relative bias on estimates was ≤ 2 % for all parameters but variance parameters. However, the biases on variance parameters significantly decreased (e.g. the bias on σγi2
 MathType@MTEF@5@5@+=feaafiart1ev1aaatCvAUfKttLearuWrP9MDH5MBPbIqV92AaeXatLxBI9gBaebbnrfifHhDYfgasaacH8akY=wiFfYdH8Gipec8Eeeu0xXdbba9frFj0=OqFfea0dXdd9vqai=hGuQ8kuc9pgc9s8qqaq=dirpe0xb9q8qiLsFr0=vr0=vr0dc8meaabaqaciaacaGaaeqabaqabeGadaaakeaaiiGacqWFdpWCdaqhaaWcbaGae83SdC2aaSbaaWqaaiabdMgaPbqabaaaleaacqaIYaGmaaaaaa@32CA@ changed from -14% to -0.4%) when increasing the number of subjects included in the sample (e.g. N = 200).

### Application

Model parameters were estimated using the HCV RNA data available during the first 4 weeks following treatment initiation in the 17 patients included in the ROCO2 trial. Among these 100 available measurements, 12 were undetectable, i.e. left-censored.

Estimates of parameters taking into account left-censoring or not are shown in Table 2. Differences between estimates according to the method used were large on the death rate infected cells, δ.

**Table 2:** Estimates of model parameters using non linear mixed models taking into account left-censored (undetectable) values (corrected) or not (crude) with data from ROCO2 clinical trial. N = 17 patients, 100 measures, 12% left-censored.

Parameter	Crude approach		Corrected approach	
	Estimate	95% CI	Estimate	95% CI
*V*_0_	6.13	5.78; 6.48	6.12	5.77; 6.47
δ	0.13	0.11; 0.17	0.19	0.14; 0.26
c	1.73	1.15; 2.61	1.66	0.88; 3.13
ε	0.89	0.74; 0.96	0.86	0.64; 0.95
σγ02 MathType@MTEF@5@5@+=feaafiart1ev1aaatCvAUfKttLearuWrP9MDH5MBPbIqV92AaeXatLxBI9gBaebbnrfifHhDYfgasaacH8akY=wiFfYdH8Gipec8Eeeu0xXdbba9frFj0=OqFfea0dXdd9vqai=hGuQ8kuc9pgc9s8qqaq=dirpe0xb9q8qiLsFr0=vr0=vr0dc8meaabaqaciaacaGaaeqabaqabeGadaaakeaaiiGacqWFdpWCdaqhaaWcbaGae83SdC2aaSbaaWqaaiabicdaWaqabaaaleaacqaIYaGmaaaaaa@325D@	0.42	0.082; 0.75	0.41	0.077; 0.74
σγ12 MathType@MTEF@5@5@+=feaafiart1ev1aaatCvAUfKttLearuWrP9MDH5MBPbIqV92AaeXatLxBI9gBaebbnrfifHhDYfgasaacH8akY=wiFfYdH8Gipec8Eeeu0xXdbba9frFj0=OqFfea0dXdd9vqai=hGuQ8kuc9pgc9s8qqaq=dirpe0xb9q8qiLsFr0=vr0=vr0dc8meaabaqaciaacaGaaeqabaqabeGadaaakeaaiiGacqWFdpWCdaqhaaWcbaGae83SdC2aaSbaaWqaaiabigdaXaqabaaaleaacqaIYaGmaaaaaa@325F@	3.23	0.38; 6.09	3.77	0.38; 7.16
σe2 MathType@MTEF@5@5@+=feaafiart1ev1aaatCvAUfKttLearuWrP9MDH5MBPbIqV92AaeXatLxBI9gBaebbnrfifHhDYfgasaacH8akY=wiFfYdH8Gipec8Eeeu0xXdbba9frFj0=OqFfea0dXdd9vqai=hGuQ8kuc9pgc9s8qqaq=dirpe0xb9q8qiLsFr0=vr0=vr0dc8meaabaqaciaacaGaaeqabaqabeGadaaakeaaiiGacqWFdpWCdaqhaaWcbaGaemyzaugabaGaeGOmaidaaaaa@30E8@	0.071	0.045; 0.097	0.073	0.044; 0.10

Using the crude approach, the estimate was 0.13 day^-1 ^(95% confidence interval [CI]: 0.11; 0.17) compared to 0.19 day^-1 ^(CI: 0.14; 0.26) when taking into account left-censoring. These estimates correspond to half-life (t_1/2_) of infected cells of 5.3 days (t_1/2 _= ln(2)/δ) and 3.6 days, respectively. Differences between estimates for the other fixed parameters were less important. Furthermore, the confidence intervals of the estimates were larger when taking into account left-censoring (Table 2).

**Figure 1:**
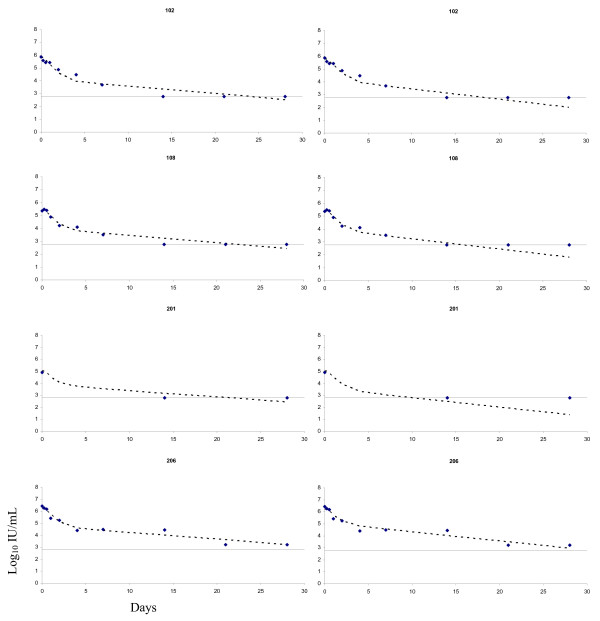
Observed and predicted HCV RNA values in four patients. Predictions came from non linear mixed effect models taking into account left-censored (undetectable) values (right side) or not (left side). Observed undetectable HCV RNA measures (<2.78 log_10 _IU/mL) are plotted at 2.78 log_10 _IU/mL.

The impact of the method used to estimate the parameters on individual viral load predictions is illustrated in Figure 1. For the first three patients (102, 108 and 201), the decrease of the second part of the viral load slope was more pronounced when taking into account left-censoring. Actually, left-censoring tended to occur on the last measurements depending on the treatment efficacy and the baseline level of viral load. The apparent discrepancy with observed values is obviously due to the fact that undetectable values are plotted on the detection limit (2.78 IU/mL) although the true value is below this threshold. This result is expected as the slope after the shoulder is proportional to the infected cell death rate (δ). As expected for the last patient (206) who did not have any undetectable viral load within 4 weeks, both predictions were very close.

The relative differences between estimates of individual treatment efficacy (ε_i _= ε + γ_1i_) according to the method used varied from 0.001% to 37%. As expected from simulation results where treatment efficacy tended to be overestimated with the crude approach and from the estimate of the average (fixed) effect ε, the estimated effect was most often higher with the crude approach compared to the corrected one. For instance, the estimate of treatment efficacy in patient 101, was 36% and 45% when taking into account left-censoring or not, respectively. On the contrary, for the patient 201, the estimates were 97% and 93%, respectively. Of note, the model was able to predict viral load changes for the patient 201 thanks to the information provided by the other patients with more numerous measurements available. This is an illustration of the advantage of hierarchical models.

## Discussion

In this paper, the impact of taking into account left-censored (undetectable) HCV RNA values was illustrated on the estimation of dynamical models based on a system of differential equations. Although, the proportion of undetectable values was quite low (12%), there were clinically significant differences, particularly in estimate of mean half-life and individual treatment efficacy. Such a result is important because all these parameters are of interest. Treatment efficacy evaluation through dynamical model is broadly used in HCV infection for instance.

We observed smaller biases from the crude approach applied to the real dataset compared to simulation results. However, some parameters values were different to those used in the simulations such as δ (0.13 vs. 0.40). Simulations using values estimated with real data led to smaller biases as observed in the present application (data not shown). The overestimation of the treatment efficacy by the crude approach may appear counter-intuitive because the imputation of the value of the threshold artificially limits the decrease of viral load. However, it is difficult to anticipate the impact of left-censoring in dynamical models because of the complex relationship between parameters, particularly between ε and δ [23]. In the present study, the imputation of the value of threshold level to undetectable viral loads led to a higher level of HCV RNA than the truth, particularly in the second part of the dynamics. The death rate of infected cells (δ) is one of the main parameters influencing viral load levels in this period [5,25]. This explains the underestimation of this parameter. On the other hand, an overestimation of treatment effect on viral production (ε) is needed to obtain a trajectory compatible with the first part of the viral dynamics (high viral load without left-censored measures), given a high infected cells death and virions clearance.

Half-life of infected cells helps in understanding how high is the turnover [3,26,27]. Previously published results [25] can be used to illustrate the size of the impact of left-censoring on HIV infected cells turnover. Differences in estimates of half-life of infected long-lived cells as large as those we reported in HCV would lead to halve the time needed to treat to achieve virus eradication (assuming no viral reservoir). Compared to results with piecewise linear mixed models commonly used with surveillance data (monthly to 6-months intervals between measurements) of HIV RNA [19,20], the estimates of the parameters are more sensitive to undetectable values in the context of dynamical models with highly repeated measurements. Moreover, confidence intervals of estimates were larger when taking into account left-censoring compared to simple imputation that tends to artificially decrease the variability, as previously reported with linear models [20].

The method presented in this paper is easy to implement in standard software. One limitation is that it is based on analytical solutions of the system of differential equations. However, looking at the applied papers on HCV infection, the authors used most often the same model with the same assumptions leading to the same analytical solution. Using hierarchical models taking into account left-censoring should improve the validity of estimation and may help in case of convergence difficulty when using individual data [9]. More complex mathematical models have been proposed to fit additional markers such as liver enzymes level [28] or pharmacokinetics data [29]. In this case, more general approaches based directly on numerical solution of the differential equations should be used [13,15]. Another limitation of the proposed methods is the assumption of log-normal distribution of viral load measures. In our experience, it is most often a reasonable assumption in the case of circulating HIV virus and this could be checked from residuals [20,30]. However, if this assumption is not tenable, extensions based on mixture distributions (log-normal and binary) can be used and are also easily implementable in software [21].

## Conclusion

Imputing a single value to left-censored measures of viral load is a wrong assumption and stronger than assuming a given distribution for the whole measurements. We proposed a method that gives unbiased estimates if the assumed distribution is correct (e.g. lognormal) and that is easy to use with standard software.

## Competing interests

The trial was supported by a grant from Roche Laboratories.

## Authors' contributions

RT carried out the simulations and drafted the manuscript. JG participated to the work of estimation (with RT). JG, HJG and DC participated in the statistical analysis and helped to draft the manuscript. GC, DN and PT performed the clinical trial and helped to draft the manuscript. All authors read and approved the final manuscript.

## Appendix

Example of code using NLMIXED to fit the model presented in the methods section taking into account left censoring.

**proc nlmixed **data = roco2 OPTCHECK;/* option for checking convergence at the optimum */

/* declare the model parameters to estimate */

   parms beta0 = **10 **beta1 = -**1.0 **beta2 = **1 **beta3 = **0.8 **s2b0 = **1 **s2b3 = **0.1 **s2 = **0.1**;

/* declare constraints for variance parameters */

   bounds s2,s2b0,s2b3 > **0**;

pi = 2*arsin(**1**);

/* model definition */

      V0 = exp(beta0+b0);

      d = exp(beta1);

      c = exp(beta2);

      e = beta3+b3;

      t0 = **0.25**;/* 6 hours */

      th = sqrt((c-d)*(c-d)+4*(**1**-e)*c*d);

      l1 = 0.5*(c+d+th);

      l2 = 0.5*(c+d-th);

      if tps le t0 then pred = V0;

      if tps gt t0 then

      pred = 0.5*V0*((**1**-(c+d-2*e*c)/th) * exp(-l1*((tps-t0)))+

         (**1**+(c+d-2*e*c)/th) * exp(-l2*((tps-t0))));

      logpred = log10(pred);

/* likelihood contribution according to the observed/censored status */

* observed ;

if detec = **1 **then ll = (**1**/(sqrt(2*pi*s2)))

         *exp(-(logCV-logpred)**2/(2*s2));

* censored ;

if detec = **0 **then ll = probnorm((logCV-logpred)/sqrt(s2));

L = log(ll);

      model logCV ~ general(L);

/* definition of the random effects */

      random b0 b3 ~ normal([**0**,**0**], [s2b0,**0**,s2b3]) subject = id;

Example of code used for simulating data from dynamical model

%do sim = **1 **%to &S;

%do id = **1 **%to &N;

Data_null_;

logCV0 = **6.16**+0.70*rannor(-**1**);

CV0 = 10**(logCV0);call symput('CV0',CV0);

kmax = **1.39**+1.64*rannor(-**1**);

e = exp(kmax)/(**1**+exp(kmax));call symput('e',e);

run;

Data sim; do time = **0 **to **672 **by **1**;output;end; run;

Proc model data = sim;

dependent T I CV ;

parm b **0.00000003 **d **0.0167 **e &e p **4.16 **c **0.0833**;

if time = **0 **then do;

CV = &CV0;

T = (c*d)/(p*b);

I = (c*CV)/p;

      end;

if time ne **0 **then do;

dert.T = **0**;

dert.I = b*CV*T-d*I;

dert.CV = (**1**-e)*p*I-c*CV;

end;

solve T I CV/dynamic out = simul;

run ; quit;

Data pat;set simul;tps = time/**24**;id = &id;CV0 = &CV0;e = &e;

if round(time) in (**0**,**6**,**12**,**24**,**48**,**96**,**168**,**336**,**504**,**672**);run;

%if &id = **1 **%then %do;

Data file; set pat;error = 0.2*rannor(-**1**);if CV gt **0 **then logCV = log10(CV)+error;run;

%end;

%else %do;

Data file; set file pat;error = 0.2*rannor(-**1**);if CV gt **0 **then logCV = log10(CV)+error;run;

%end;

/* truncation */

Data file; set file;

      if logCV lt **2.778 **then do;

         logCV = **2.778**;detec = **0**;end;

      else detec = **1**;

run;

ods exclude none;

%end;/* end of patients */

## Pre-publication history

The pre-publication history for this paper can be accessed here:


